# Integrated Bioinformatics and Machine Learning Analysis Identifies Inflammation-Related Biomarkers and Immune Infiltration Patterns in Atherosclerosis

**DOI:** 10.3390/genes17070830

**Published:** 2026-07-21

**Authors:** Le Zhang, Yu Liu

**Affiliations:** 1 Department of Biochemistry and Molecular Biology, College of Pharmacy, Hebei University of Chinese Medicine, Shijiazhuang 050000, China; 2 Hebei Key Laboratory of Chinese Medicine Research on Cardio-Cerebrovascular Disease, Shijiazhuang 050091, China; 3 Hebei Higher Education Institute Applied Technology Research Center on TCM Development and Industrialization, Shijiazhuang 050091, China

**Keywords:** atherosclerosis, inflammation-related genes, immune infiltration, machine learning, diagnostic biomarkers

## Abstract

**Background:** Atherosclerosis (AS) is a chronic inflammatory vascular disease lacking reliable biomarkers for early diagnosis and risk stratification. This study aimed to identify hub genes with diagnostic potential and characterize immune microenvironment remodeling in AS. **Methods:** GSE43292 and GSE100927 were integrated as the training cohort (*n* = 168), while GSE41571, GSE120521, and GSE28829 served as independent validation cohorts (*n* = 48). Batch effects were corrected using the ComBat algorithm. Differentially expressed genes (DEGs) were identified using limma, followed by GO/KEGG enrichment analysis. LASSO regression and Random Forest analysis were performed to identify hub genes. A logistic regression diagnostic model was constructed and evaluated using ROC analysis, 5-fold cross-validation, and external validation. Immune infiltration was assessed using ssGSEA, and correlations between hub genes and immune cells were analyzed using Spearman correlation. **Results:** A total of 1349 DEGs (870 upregulated and 479 downregulated) were identified. GO and KEGG analyses demonstrated significant enrichment of immune- and inflammation-related biological processes and pathways. Seven hub genes (IBSP, XAF1, SCAMP5, SAMD9L, MYBL1, PCDH12, and CDH19) were identified through the combined application of LASSO regression and Random Forest analysis. The 7-gene logistic model achieved excellent performance in the training cohort (AUC = 0.992, 95% CI: 0.981–1.000), with a mean 5-fold cross-validation AUC of 0.984 ± 0.014, and maintained robust performance in three independent validation cohorts (AUC range: 0.952–1.000). Immune infiltration analysis revealed extensive immune microenvironment remodeling, with significantly increased infiltration of 22 of the 24 immune cell types, particularly monocytes, macrophages, and myeloid cells. Spearman correlation analysis demonstrated strong associations between hub genes, particularly SAMD9L and IBSP, and immune cell infiltration. **Conclusions:** This study identified a robust 7-gene diagnostic signature for AS and revealed its close association with the inflammatory immune microenvironment, providing potential biomarkers for early diagnosis and risk stratification.

## 1. Introduction

Atherosclerosis (AS) is a chronic and progressive inflammatory vascular disease [1]. Its major pathological features include lipid accumulation within the arterial wall, endothelial dysfunction, vascular smooth muscle cell proliferation, and inflammatory cell infiltration [2]. These pathological alterations gradually promote arterial lumen narrowing, plaque formation, and plaque rupture, ultimately leading to severe cardiovascular events such as myocardial infarction, stroke, and peripheral arterial disease [3]. In recent years, driven by population aging and lifestyle changes, the global incidence and prevalence of AS have continued to rise, making it one of the leading causes of morbidity and mortality worldwide [4]. Epidemiological studies have shown that cardiovascular diseases account for approximately 17.9 million deaths annually, representing nearly 32% of all deaths worldwide, with the majority being closely associated with AS [5]. Although current therapeutic strategies, including statin-based lipid-lowering therapy, antiplatelet treatment, and revascularization procedures, have significantly improved clinical outcomes, a substantial proportion of patients still experience residual cardiovascular risk. This finding suggests that the molecular mechanisms underlying AS remain incompletely understood and that existing preventive and therapeutic approaches still have important limitations [6]. Therefore, elucidating the molecular pathogenesis of AS and identifying key genes with potential value for early diagnosis and risk assessment are of considerable scientific significance and clinical relevance for improving the prognosis of patients with AS.

Currently, the clinical diagnosis of AS mainly depends on imaging techniques, such as vascular ultrasound, computed tomography angiography, and magnetic resonance imaging, combined with the assessment of traditional cardiovascular risk factors including hypertension, hyperlipidemia, diabetes mellitus, and smoking [7,8]. However, these methods remain insufficient for the early detection of AS, assessment of plaque vulnerability, and prediction of cardiovascular risk. Imaging examinations usually detect lesions only after plaque formation or hemodynamic changes have occurred, limiting their value in early diagnosis [9]. In addition, AS is a highly heterogeneous disease characterized by marked differences in plaque phenotype, inflammatory status, and disease progression among individuals, while reliable biomarkers reflecting its molecular pathological features are still lacking [10]. With the rapid development of high-throughput sequencing and bioinformatics, transcriptomic analysis of differentially expressed genes has become an important approach for elucidating the molecular mechanisms of AS and identifying novel diagnostic biomarkers [11]. Nevertheless, most current transcriptomic studies are based on single datasets or platforms and are therefore limited by small sample sizes, data heterogeneity, and poor reproducibility [12]. Thus, integrated analysis of multiple independent datasets may help overcome these limitations and improve the reliability of differential gene identification.

Accumulating evidence has demonstrated that inflammatory responses and dysregulation of the immune microenvironment play central roles in the initiation, progression, and plaque rupture of AS [13,14,15]. The “inflammatory hypothesis” of AS is now widely accepted, proposing that atherosclerosis is essentially a lipid-driven chronic inflammatory disease [16,17]. During the early stage of AS, endothelial cells are activated by disturbed shear stress, oxidized lipoproteins, and inflammatory mediators, leading to the upregulation of adhesion molecules and chemokines that recruit circulating monocytes into the subendothelial space [18]. These monocytes subsequently differentiate into macrophages, engulf oxidized low-density lipoprotein to form foam cells, and release various pro-inflammatory cytokines and matrix metalloproteinases, thereby amplifying local inflammatory responses and promoting plaque formation and instability [19,20]. In addition to the monocyte–macrophage lineage, multiple immune cell types, including T lymphocytes, B lymphocytes, dendritic cells, and mast cells, are deeply involved in the immune-inflammatory network of AS [21]. Among them, CD4+ T helper cells regulate inflammatory responses through the secretion of cytokines such as interferon-γ and interleukin-17, whereas regulatory T cells exert immunosuppressive effects that help limit excessive inflammatory injury [22,23]. CD8+ cytotoxic T cells and natural killer cells also contribute to endothelial injury and plaque erosion [24,25]. These immune cells interact through cytokine networks, direct cell–cell contact, and antigen presentation, collectively shaping the immune microenvironment of AS lesions. Therefore, systematic evaluation of immune cell infiltration patterns in AS tissues may provide important insights into the immunopathological mechanisms underlying AS.

With the rapid development of microarray and RNA sequencing technologies, large amounts of disease-related gene expression data have accumulated in public databases such as the Gene Expression Omnibus (GEO), providing valuable resources for bioinformatics research [26]. Integrative analysis of multiple independent datasets can effectively increase sample size, reduce batch effects, and improve statistical power, thereby facilitating the identification of more robust differentially expressed genes. Functional enrichment analyses, including GO and KEGG analyses, further enable the exploration of the biological processes and signaling pathways associated with these genes [27]. In addition, protein–protein interaction network analysis can identify key hub genes at the molecular interaction level, providing a basis for subsequent feature selection [28]. However, high-dimensional gene expression data often contain redundant and noisy features, which may lead to overfitting and poor generalizability when directly applied to diagnostic model construction. Therefore, least absolute shrinkage and selection operator (LASSO) regression is widely used for feature selection by shrinking irrelevant variables to zero and identifying core genes most closely associated with disease classification. This method improves model stability, interpretability, and potential clinical applicability. Logistic regression, as a classical interpretable classification algorithm, is commonly used to construct multigene diagnostic models [29]. Compared with single-gene biomarkers, multigene models based on integrated transcriptomic features may provide improved robustness and predictive performance for AS diagnosis and risk assessment. Moreover, combining machine learning strategies with immune infiltration analysis may facilitate the identification of biologically relevant biomarkers associated with inflammatory immune remodeling in AS. Moreover, ROC curves and the AUC are widely applied to evaluate model performance, while external validation using independent datasets is essential for assessing model generalizability and potential overfitting.

Based on the above research background and analytical strategy, this study integrated multiple atherosclerosis-related gene expression datasets from the GEO database to systematically identify differentially expressed inflammation-related genes and further determine key genes through functional enrichment and protein–protein interaction network analyses. Subsequently, machine learning approaches were applied to screen core feature genes and construct a diagnostic prediction model, which was further validated in independent datasets to assess its generalizability. In addition, immune infiltration analysis was performed to characterize the immune microenvironment of AS. Overall, this study aimed to elucidate the inflammation- and immune-related molecular characteristics of AS and to identify potential biomarkers for early diagnosis and risk stratification.

## 2. Methods

### 2.1. Data Acquisition and Batch Effect Correction

**Figure 1:**
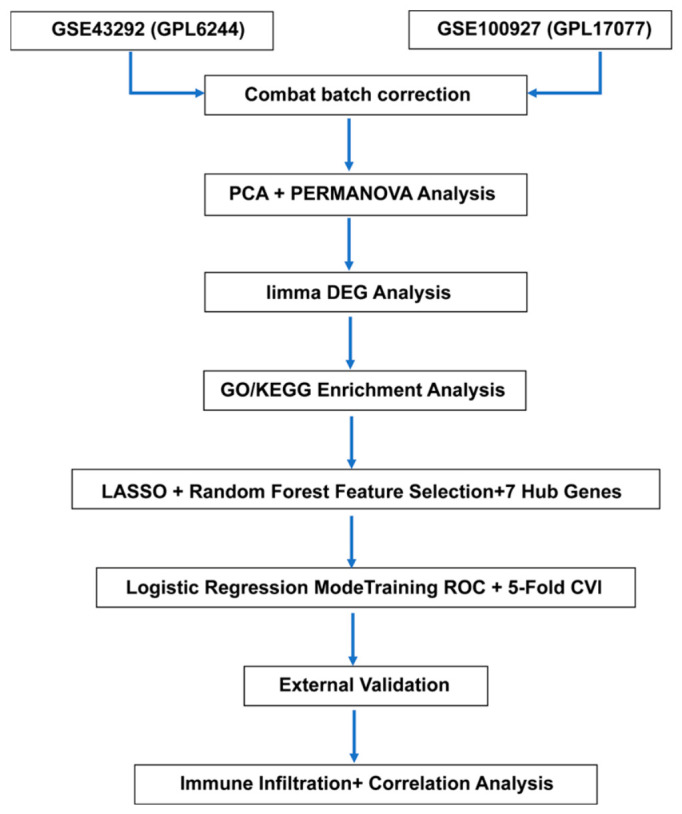
**Workflow diagram of the study.** The flowchart illustrates the overall analytical pipeline, including data acquisition (GSE43292 and GSE100927), ComBat batch correction, PCA with PERMANOVA analysis, limma differential expression analysis, GO/KEGG enrichment, LASSO and Random Forest feature selection, logistic regression model construction and validation (ROC curve + 5-fold cross-validation), external validation, and immune infiltration with correlation analysis.

The overall workflow of the present study is illustrated in Figure 1. Gene expression datasets related to AS were downloaded from the GEO database, including two training datasets (GSE43292 and GSE100927) and three independent validation datasets (GSE41571, GSE120521, and GSE28829). GSE43292, generated on the GPL6244 platform, included 32 atherosclerotic plaque samples and 32 adjacent normal tissue samples, whereas GSE100927, generated on the GPL17077 platform, contained 69 atherosclerotic plaque samples and 35 normal control samples.

Raw expression matrices were imported and preprocessed using Python (pandas, version 2.0.3). A total of 17,573 genes shared between GSE43292 and GSE100927 were retained, and the two datasets were subsequently merged. Batch effects introduced by different microarray platforms were corrected using the ComBat algorithm implemented in the sva package (version 3.58.0) in R. ComBat employs an empirical Bayes framework to estimate batch-specific effects while incorporating the AS/Control grouping variable as a covariate, thereby minimizing technical variation while preserving biological differences between groups.

After batch correction, the merged training cohort comprised 168 samples, including 101 AS samples and 67 control samples. Detailed information on the training datasets, including the microarray platforms and sample sizes, is summarized in Supplementary Table S1.

The three independent validation cohorts (GSE41571, GSE120521, and GSE28829) were preprocessed using the same pipeline as the training cohort, including log_2_ transformation, probe annotation, gene mapping, and normalization. GSE41571 (GPL570) included 6 stable plaques and 5 ruptured plaques, GSE120521 (GPL11154) comprised 4 stable plaques and 4 unstable plaques, and GSE28829 (GPL570) consisted of 13 early-stage plaques and 16 advanced plaques. Collectively, these three validation cohorts comprised 48 samples and were analyzed separately for external validation of the diagnostic model.

### 2.2. Principal Component Analysis

To evaluate the effectiveness of ComBat batch correction and the overall sample distribution of the training cohort, principal component analysis (PCA) was performed on the corrected expression matrix. The first three principal components were calculated using the sklearn.decomposition module in Python. PCA plots were generated with samples colored according to dataset origin (GSE43292 vs. GSE100927) to assess batch effect removal. Substantial intermixing of samples from different datasets was considered indicative of successful batch correction. PCA plots colored according to sample group (AS vs. Control) were further used to evaluate whether biological differences were preserved following batch correction. In addition, permutational multivariate analysis of variance (PERMANOVA) was performed to quantitatively assess the effectiveness of batch correction.

### 2.3. Differential Expression Analysis

Differential expression analysis between AS and control samples in the training cohort was performed using the limma package (version 3.66.0) in R. The limma algorithm applies empirical Bayes moderation to the *t*-statistics, thereby improving the stability and reliability of differential expression analysis, particularly for datasets with relatively small sample sizes. Genes satisfying the criteria of |log_2_FC| > 0.5 and Benjamini–Hochberg false discovery rate (FDR) < 0.05 were considered differentially expressed genes (DEGs). The log_2_ fold change (log_2_FC) represents the logarithm (base 2) of the relative expression level between AS and control samples.

The identified DEGs were subsequently used for GO and KEGG enrichment analyses, LASSO regression, Random Forest feature selection, and all downstream analyses.

### 2.4. Visualization Analysis of DEGs

To visualize the overall distribution and expression patterns of DEGs, volcano plots and M–A (MA) plots were generated. Volcano plots illustrate the statistical significance and magnitude of gene expression changes, whereas MA plots depict the relationship between mean expression levels and log_2_ fold changes, reflecting the global distribution of gene expression differences.

In addition, heatmaps of the top 15 DEGs ranked by adjusted *p*-value were generated based on Z-score-normalized expression matrices. Hierarchical clustering analysis was performed to evaluate expression patterns across both samples and genes.

### 2.5. Functional Enrichment Analysis Methods

GO and KEGG enrichment analyses were performed on the identified DEGs using the clusterProfiler package (version 4.10.0) in R. GO analysis included three categories: biological process (BP), cellular component (CC), and molecular function (MF).

Statistical significance of enrichment was assessed using Fisher’s exact test, followed by Benjamini–Hochberg correction for multiple testing. Terms with FDR < 0.05 were considered significantly enriched. KEGG pathway enrichment analysis was conducted using the same statistical framework. Significantly enriched GO terms were visualized using bubble plots, whereas significantly enriched KEGG pathways were visualized using bar plots.

### 2.6. LASSO Regression and Random Forest Feature Selection

To identify robust diagnostic biomarkers for atherosclerosis, machine learning-based feature selection was performed using LASSO regression followed by Random Forest analysis. LASSO regression was implemented using the glmnet package (version 4.1.8) in R. The optimal regularization parameter (λ) was determined by 5-fold cross-validation, and the lambda.1se criterion was selected to obtain a more parsimonious feature set. Consequently, 16 candidate genes with non-zero coefficients were retained (MYBL1, IBSP, SAMD9L, XAF1, CDH19, SNORD116-4, SCAMP5, EGR2, CX3CR1, PCDH12, HLA-DOB, GPC3, SMPX, CLTC-IT1, NR1D1, and SERPINA3).

These 16 candidate genes were subsequently subjected to Random Forest analysis using the randomForest package (version 4.7-1). Gene importance was evaluated according to MeanDecreaseAccuracy and MeanDecreaseGini. The top eight genes ranked by MeanDecreaseGini were retained for further evaluation. Because SNORD116-4 was unavailable across the independent validation datasets due to platform differences, it was excluded from subsequent analyses. Consequently, seven hub genes (IBSP, XAF1, SCAMP5, SAMD9L, MYBL1, PCDH12, and CDH19) were retained for diagnostic model construction, external validation, and immune infiltration analysis.

### 2.7. Methods for Diagnostic Model Construction and Evaluation

A diagnostic model was constructed using logistic regression based on the seven hub genes (IBSP, XAF1, SCAMP5, SAMD9L, MYBL1, PCDH12, and CDH19). Model fitting was performed using the glm function in R, and regression coefficients were estimated by maximum likelihood estimation. The model was defined as follows:

P(Y = 1|X) = 1/(1 + e^−(β0 + ΣβjXj)^)

where P(Y=1∣X) represents the probability of a sample being classified as AS, $\beta_{0}$ is the intercept, and $\beta_{i}$ represents the regression coefficient of each hub gene.

Diagnostic performance was evaluated using receiver operating characteristic (ROC) curves and the area under the ROC curve (AUC). Sensitivity, specificity, and the optimal cutoff determined by the Youden index were also calculated. Model robustness was assessed by 5-fold cross-validation within the training cohort and further validated in three independent validation cohorts (GSE41571, GSE28829, and GSE120521). The 95% confidence intervals (CIs) of the AUCs were calculated using the DeLong method.

The results of the 5-fold cross-validation were visualized using boxplots and bar plots to illustrate the distribution and consistency of AUC values across folds.

### 2.8. Immune Infiltration Analysis

To characterize the immune microenvironment of atherosclerosis, single-sample gene set enrichment analysis (ssGSEA) was performed to quantify immune cell infiltration levels in the training cohort using the GSVA package (version 1.50.0) in R. A total of 24 immune cell types were evaluated, including T cells (CD4^+^ T, CD8^+^ T, Treg, Th1, Th2, Th17, Tfh, and γδ T cells), B cells, plasma cells, NK cells, NKT cells, macrophages (M1 and M2), monocytes, dendritic cells (DCs, pDCs, and mDCs), neutrophils, eosinophils, mast cells, lymphocytes, and myeloid cells. Gene signatures for immune cell types were obtained from the study by Charoentong et al. [30] The gene signatures used to define the 24 immune cell types are summarized in Supplementary Table S2.

Based on the ssGSEA-derived infiltration scores, heatmaps, bar plots, and boxplots were generated to characterize immune infiltration patterns and compare differences between the AS and control groups. Group comparisons were performed using the Mann–Whitney U test, followed by Benjamini–Hochberg FDR correction. FDR < 0.05 was considered statistically significant.

### 2.9. Correlation Analysis Between Hub Genes and Immune Cell Infiltration

To further investigate the relationship between hub genes and the immune microenvironment, Spearman rank correlation analysis was performed between the expression levels of the seven hub genes and the infiltration scores of 24 immune cell types. The Spearman correlation coefficients (ρ) and corresponding *p* values were calculated using the stats package in R, followed by Benjamini–Hochberg FDR correction for multiple testing. Only correlations satisfying |ρ| ≥ 0.5 and FDR < 0.05 were retained and visualized using a heatmap.

### 2.10. Statistical Analysis

All statistical analyses were performed using R software (version 4.5.3; R Foundation for Statistical Computing, Vienna, Austria). Comparisons between two groups of continuous variables were conducted using the Mann–Whitney U test. Correlation analyses were performed using Spearman’s rank correlation. All statistical tests were two-sided, and *p* < 0.05 was considered statistically significant. For analyses involving multiple comparisons, *p* values were adjusted using the Benjamini–Hochberg false discovery rate (FDR) method. A summary of all statistical methods is provided in Supplementary Table S3.

## 3. Results

### 3.1. Results of Principal Component Analysis

**Figure 2:**
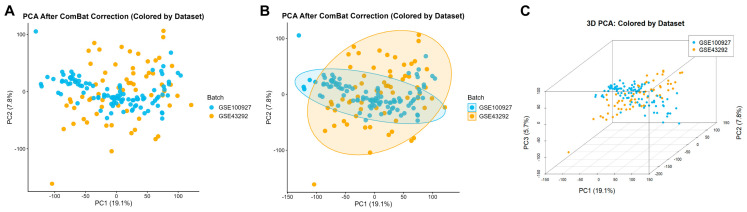
**Principal component analysis (PCA) of the merged training datasets after ComBat batch effect correction.** Principal component analysis (PCA) of the ComBat-corrected training cohort colored according to dataset origin (GSE43292 and GSE100927). (**A**) Two-dimensional PCA plot. (**B**) Two-dimensional PCA plot with 95% confidence ellipses. (**C**) Three-dimensional PCA plot. PC1 and PC2 explained 19.1% and 7.8% of the total variance, respectively. Samples from the two datasets showed substantial overlap after ComBat correction. PERMANOVA analysis revealed no significant difference between GSE43292 and GSE100927 (F = 0.8103, *p* = 0.558; 999 permutations), indicating effective removal of batch effects.

To evaluate the effectiveness of batch correction and the overall distribution of the training cohort, PCA was performed on the ComBat-corrected expression matrix. As shown in Figure 2A, samples from GSE43292 and GSE100927 were substantially intermixed after ComBat correction. The 95% confidence ellipses further demonstrated considerable overlap between the two datasets (Figure 2B), indicating effective removal of batch effects. The three-dimensional PCA plot (Figure 2C) further confirmed the mixing of samples from different datasets. PC1 and PC2 explained 19.1% and 7.8% of the total variance, respectively.

PERMANOVA analysis showed no significant difference between GSE43292 and GSE100927 after ComBat correction (F = 0.8103, *p* = 0.558, 999 permutations), further supporting the effectiveness of batch correction. In addition, PCA with samples colored according to biological group (Control vs. AS) showed clear separation between the two groups (Supplementary Figure S1). PERMANOVA analysis also confirmed a significant difference between AS and Control samples (F = 34.5244, *p* = 0.001, 999 permutations), indicating that biological differences were preserved after batch correction. These results demonstrated that the ComBat correction effectively minimized batch effects while preserving biological variation, supporting the suitability of the training cohort for subsequent differential expression analysis and machine learning modeling.

### 3.2. Identification of Differentially Expressed Genes

**Figure 3:**
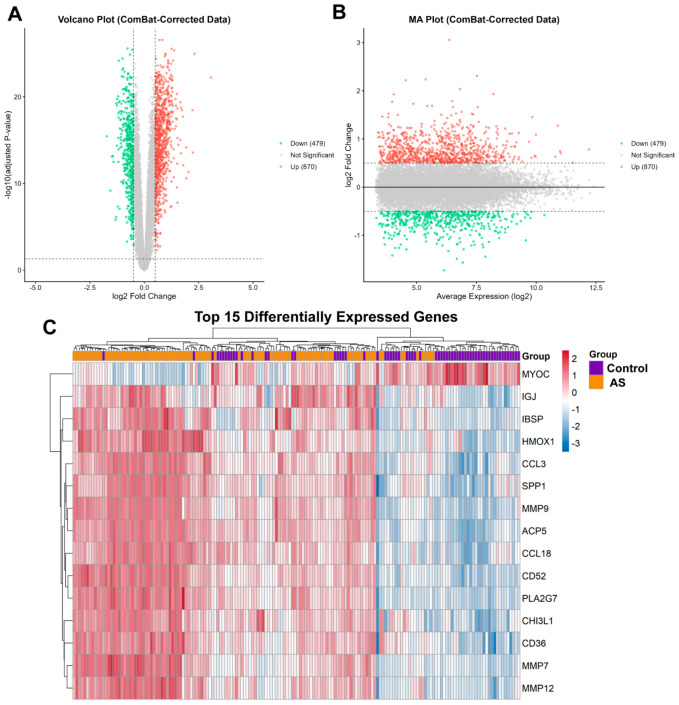
**Differential expression analysis of the ComBat-corrected training cohort.** (**A**) Volcano plot showing differentially expressed genes (DEGs) between AS and control samples. Red dots represent upregulated genes, green dots represent downregulated genes, and gray dots represent non-significant genes. The horizontal dashed line indicates the significance threshold (*p* = 0.05). (**B**) MA plot showing the relationship between the average gene expression level and log_2_ fold change, with significantly upregulated and downregulated genes highlighted. The horizontal dashed lines indicate the log2 fold change threshold used for differential expression analysis. (**C**) Heatmap of the top 15 differentially expressed genes across all samples in the training cohort. Each row represents a gene and each column represents a sample. Expression values were Z-score normalized, with red indicating relatively high expression and blue indicating relatively low expression. Samples are annotated by group at the top of the heatmap, with purple representing the Control group and yellow representing the AS group.

Differential expression analysis between AS and Control samples was conducted using the limma package with empirical Bayes moderation. A total of 1349 differentially expressed genes (DEGs) were identified using the thresholds of |log_2_FC| > 0.5 and adjusted *p* < 0.05 (Benjamini–Hochberg FDR correction), including 870 upregulated and 479 downregulated genes in AS samples compared with Controls. The top 15 DEGs ranked by adjusted *p* value are summarized in Table 1. Among the top-ranked DEGs, MMP9 (log_2_FC = 3.056, adjusted *p* = 5.99 × 10^−23^), IBSP (log_2_FC = 2.311, adjusted *p* = 1.08 × 10^−25^), and MMP7 (log_2_FC = 2.234, adjusted *p* = 2.68 × 10^−14^) were the most significantly upregulated genes, whereas MYOC (log_2_FC = −1.726, adjusted *p* = 3.69 × 10^−16^) was the most significantly downregulated gene. The volcano plot and MA plot illustrated the overall distribution of DEGs and their expression changes (Figure 3A,B). The heatmap of the top 15 DEGs further demonstrated distinct expression patterns between AS and Control samples, with hierarchical clustering effectively separating the two groups (Figure 3C).

The consistency of differential expression between the two training datasets was further evaluated by comparing DEGs identified independently in GSE43292 and GSE100927. As shown in Supplementary Figure S2, 1098 DEGs were identified in GSE43292 and 1868 DEGs were identified in GSE100927, with 719 shared DEGs, accounting for 65.5% of the DEGs identified in GSE43292. A summary of the DEG overlap analysis is provided in Supplementary Table S4. These findings indicate that differential expression patterns were highly reproducible across the two independent training cohorts.

### 3.3. Functional Enrichment Analysis

**Figure 4:**
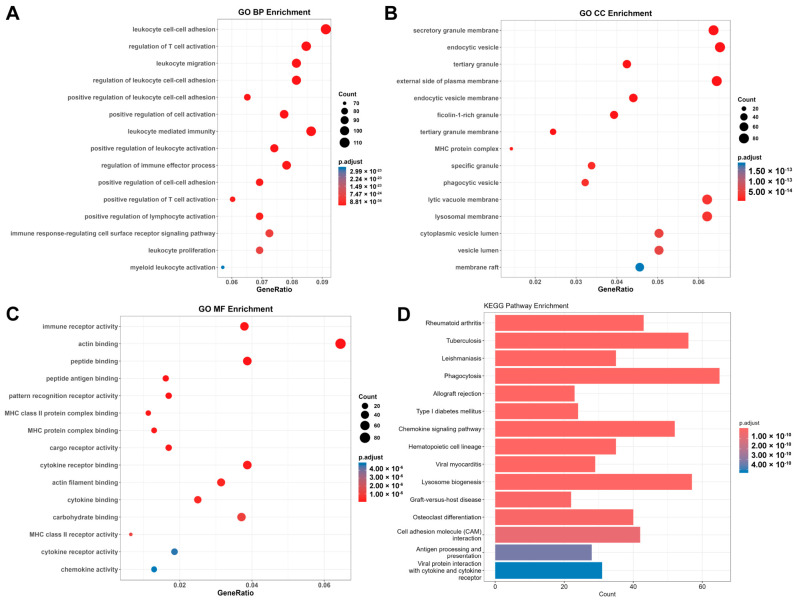
**Functional enrichment analysis of differentially expressed genes (DEGs) in the training cohort.** Functional enrichment analysis of differentially expressed genes (DEGs) identified in the ComBat-corrected training cohort. (**A**) Top enriched Gene Ontology biological process (GO-BP) terms. (**B**) Top enriched Gene Ontology cellular component (GO-CC) terms. (**C**) Top enriched Gene Ontology molecular function (GO-MF) terms. (**D**) Top enriched Kyoto Encyclopedia of Genes and Genomes (KEGG) pathways. The x-axis represents the gene ratio, and the dot size indicates the number of enriched genes. Dot color represents the adjusted *p* value, with darker red indicating greater statistical significance.

Gene Ontology (GO) and Kyoto Encyclopedia of Genes and Genomes (KEGG) enrichment analyses were performed to investigate the biological functions and signaling pathways associated with the identified differentially expressed genes (DEGs). GO enrichment analysis demonstrated that the DEGs were predominantly enriched in immune- and inflammation-related biological processes, including leukocyte cell–cell adhesion, regulation of T cell activation, leukocyte migration, positive regulation of leukocyte activation, and leukocyte-mediated immunity (Figure 4A, FDR < 0.05). In the cellular component (CC) category, the DEGs were mainly enriched in secretory granule membrane, endocytic vesicle, external side of the plasma membrane, lysosomal membrane, and cytoplasmic vesicle lumen (Figure 4B). Molecular function (MF) analysis further revealed significant enrichment in immune receptor activity, cytokine binding, cytokine receptor binding, peptide binding, and pattern recognition receptor activity (Figure 4C).

KEGG pathway analysis identified multiple significantly enriched pathways associated with immune regulation and inflammatory responses, including phagocytosis, chemokine signaling pathway, osteoclast differentiation, cell adhesion molecules (CAMs), lysosome, and rheumatoid arthritis (Figure 4D, FDR < 0.05). These enrichment results indicate that dysregulated immune activation, inflammatory signaling, leukocyte migration, and innate immune responses are closely associated with the pathogenesis of AS.

### 3.4. Identification of Hub Genes by Machine Learning

**Figure 5:**
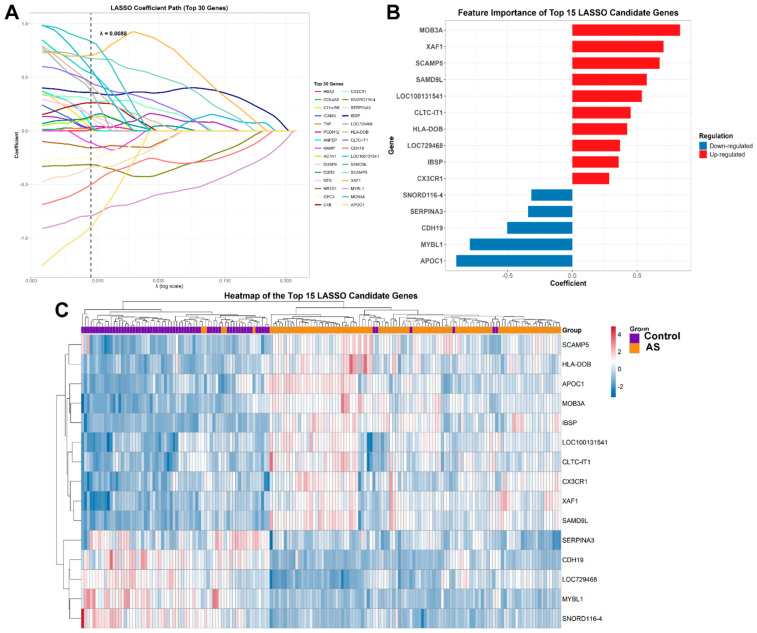
**Identification of candidate genes using least absolute shrinkage and selection operator (LASSO) regression in the ComBat-corrected training cohort.** (**A**) LASSO coefficient profiles of the top 30 candidate genes. The x-axis represents log(λ), and the y-axis represents regression coefficients. The vertical dashed line indicates the selected lambda.1se value used to obtain the final 16 candidate genes. Only the top 30 genes with the largest absolute coefficients are displayed for visualization. (**B**) Regression coefficients of the top 15 candidate genes selected by the LASSO model. Positive coefficients (red) indicate genes upregulated in AS, whereas negative coefficients (blue) indicate genes downregulated in AS. (**C**) Heatmap showing the expression patterns of the top 15 LASSO candidate genes across all samples in the training cohort. Each row represents a gene and each column represents a sample. Expression values were Z-score normalized, with red indicating relatively high expression and blue indicating relatively low expression. Samples are annotated by group at the top of the heatmap, with purple representing the Control group and yellow representing the AS group.

LASSO regression analysis was performed on the 1349 differentially expressed genes (DEGs) to identify robust diagnostic biomarkers for AS. The optimal regularization parameter (λ) was determined using 5-fold cross-validation, and the lambda.1se criterion was selected to construct a more parsimonious model. Using the selected lambda.1se value, 16 candidate genes with non-zero coefficients were retained, including MYBL1, IBSP, SAMD9L, XAF1, CDH19, SNORD116-4, SCAMP5, EGR2, CX3CR1, PCDH12, HLA-DOB, GPC3, SMPX, CLTC-IT1, NR1D1, and SERPINA3. The complete list of the 30 LASSO-selected candidate genes and their corresponding coefficients is provided in Supplementary Table S5. The LASSO coefficient path plot illustrated the shrinkage of gene coefficients with increasing penalty (Figure 5A). The regression coefficients of the top 15 candidate genes are shown in Figure 5B, and the heatmap further demonstrated their distinct expression patterns between AS and Control samples (Figure 5C).

Subsequently, random Forest analysis was performed using these 16 candidate genes, and gene importance was evaluated according to MeanDecreaseAccuracy and MeanDecreaseGini (Supplementary Table S6 and Figure S4). Based on the MeanDecreaseGini ranking, the top eight candidate genes were retained. Because SNORD116-4 was unavailable across the independent validation datasets due to platform differences, it was excluded from subsequent analyses. Consequently, seven hub genes, namely IBSP, XAF1, SCAMP5, SAMD9L, MYBL1, PCDH12, and CDH19, were retained for diagnostic model construction, expression validation, external validation, and immune infiltration analysis (Table 2).

### 3.5. Construction and Evaluation of the Diagnostic Model

**Figure 6:**
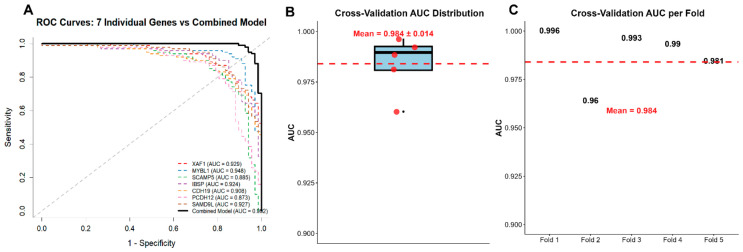
**Diagnostic performance and cross-validation of the 7-gene diagnostic model in the training cohort.** (**A**) Receiver operating characteristic (ROC) curves comparing the diagnostic performance of the seven individual hub genes and the combined logistic regression model for distinguishing AS from control samples. The area under the ROC curve (AUC) is shown for each model. (**B**) Distribution of the AUC values obtained from five-fold cross-validation of the combined diagnostic model. The boxplot summarizes the variability in model performance, and the red dashed line indicates the mean AUC. (**C**) AUC values for each fold of the five-fold cross-validation. The red dashed line represents the mean AUC across all folds, demonstrating the robustness and stability of the diagnostic model. The red dashed lines in panels (**B**,**C**) indicate the mean AUC across the five cross-validation folds.

A multivariable logistic regression model was constructed using the seven hub genes. In the training cohort, the model demonstrated excellent diagnostic performance, achieving an AUC of 0.992 (95% CI: 0.981–1.000), with a sensitivity of 0.950 and a specificity of 0.970 at the optimal cutoff value (0.765), as determined by the Youden index (Figure 6A). To evaluate the robustness of the diagnostic model, 5-fold cross-validation was performed. The model achieved a mean AUC of 0.984 (SD = 0.014), with individual fold AUC values of 0.996, 0.960, 0.993, 0.990, and 0.981. The distribution of AUC values across the five folds is shown in Figure 6B, whereas the AUC achieved in each fold is presented in Figure 6C. In addition, the calibration curve demonstrated good agreement between the predicted probabilities and the observed outcomes in the training cohort, indicating that the 7-gene logistic regression model was well calibrated (Figure S3).

### 3.6. Expression Validation of the Seven Hub Genes

**Figure 7:**
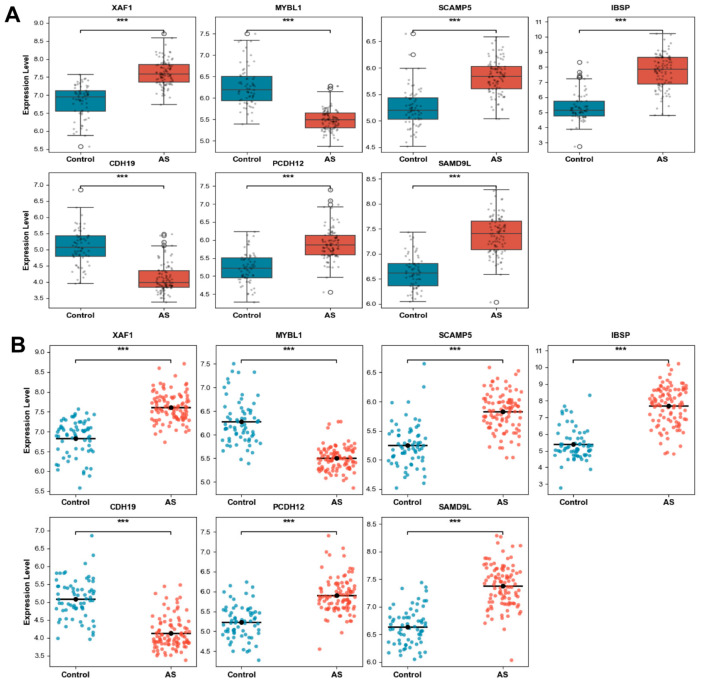
**Expression patterns of the seven hub genes in the training cohort.** Expression patterns of the seven hub genes in the ComBat-corrected training cohort. (**A**) Boxplots comparing the expression levels of the seven hub genes (XAF1, MYBL1, SCAMP5, IBSP, CDH19, PCDH12, and SAMD9L) between AS and control samples. Boxes represent the interquartile range (IQR), the center line indicates the median, whiskers represent 1.5 × IQR, and each dot represents an individual sample. (**B**) Scatter plots showing the expression distribution of the seven hub genes in individual samples. Horizontal black lines indicate the mean expression level of each group. *** *p* < 0.001.

The expression patterns of the seven hub genes identified by the machine learning pipeline were evaluated by comparing their expression levels between AS and Control samples in the training cohort. As shown in Figure 7A, all seven hub genes were differentially expressed between the two groups (all FDR < 0.001, Wilcoxon rank-sum test). Specifically, XAF1, SCAMP5, IBSP, PCDH12, and SAMD9L were significantly upregulated in AS samples, whereas MYBL1 and CDH19 were significantly downregulated compared with the Control group. Scatter plots further demonstrated the distribution of individual samples and confirmed the distinct expression patterns of the seven hub genes between the two groups (Figure 7B). These findings confirmed the differential expression patterns of the seven hub genes and further supported their potential as diagnostic biomarkers for AS.

### 3.7. External Validation of the Diagnostic Model

**Figure 8:**
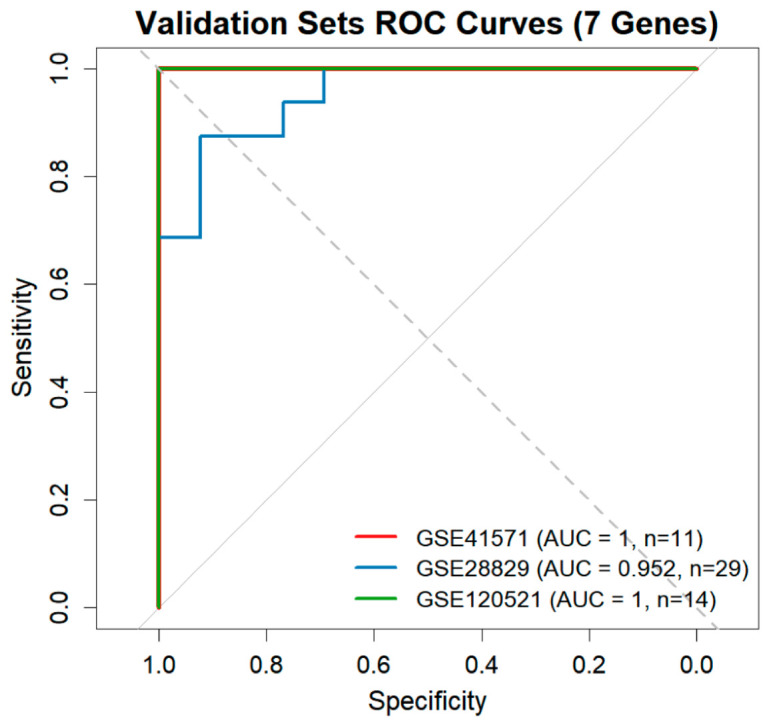
**External validation of the 7-gene diagnostic model in independent validation cohorts.** External validation of the 7-gene diagnostic model in three independent validation cohorts. Receiver operating characteristic (ROC) curves were used to evaluate the diagnostic performance of the logistic regression model based on the seven hub genes in GSE41571 (*n* = 11), GSE28829 (*n* = 29), and GSE120521 (*n* = 14). The corresponding area under the ROC curve (AUC) values were 1.000, 0.952, and 1.000, respectively, demonstrating the excellent diagnostic performance and generalizability of the model across independent cohorts. The gray dashed diagonal line represents the performance of a random classifier (AUC = 0.5).

The generalizability of the 7-gene model was evaluated in three independent cohorts (GSE41571, GSE28829, and GSE120521). As shown in Figure 8, the model achieved AUCs of 1.000 (95% CI: 1.000–1.000, *n* = 11), 0.952 (95% CI: 0.886–1.000, *n* = 29), and 1.000 (95% CI: 1.000–1.000, *n* = 14), respectively, with a mean AUC of 0.984 (Table 3). Although the validation cohorts represented different plaque phenotypes and progression stages rather than AS versus Control classification, the model consistently demonstrated high diagnostic performance across all three cohorts. Notably, the largest validation cohort (GSE28829, *n* = 29) achieved an AUC of 0.952, supporting the robustness of the diagnostic model in an independent dataset.

### 3.8. Immune Cell Infiltration Analysis

**Figure 9:**
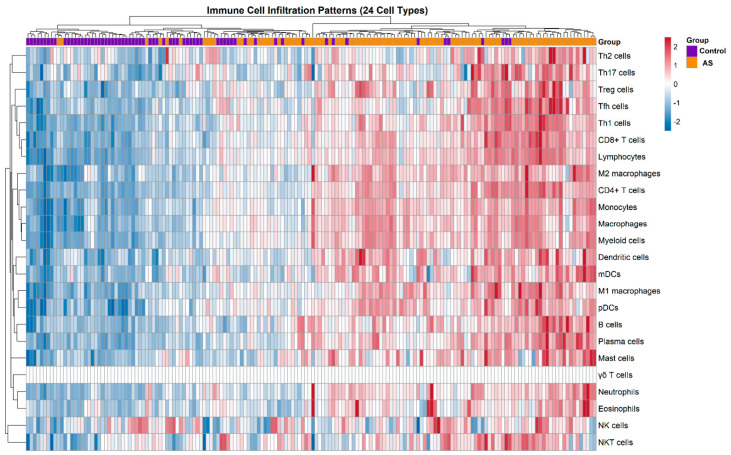
**Immune cell infiltration patterns in the training cohort.** Immune cell infiltration patterns in the ComBat-corrected training cohort estimated by single-sample gene set enrichment analysis (ssGSEA). The heatmap displays the infiltration scores of 24 immune cell types across all samples. Each row represents an immune cell type, and each column represents an individual sample. Infiltration scores were Z-score normalized, with red indicating relatively high infiltration and blue indicating relatively low infiltration. Samples are annotated by group at the top of the heatmap, with purple representing the Control group and yellow representing the AS group.

The immune landscape of the training cohort was characterized by quantifying the infiltration levels of 24 immune cell types using single-sample gene set enrichment analysis (ssGSEA). As shown in Figure 9, AS samples generally exhibited higher immune infiltration levels than Control samples, indicating an enhanced inflammatory immune microenvironment.

**Figure 10:**
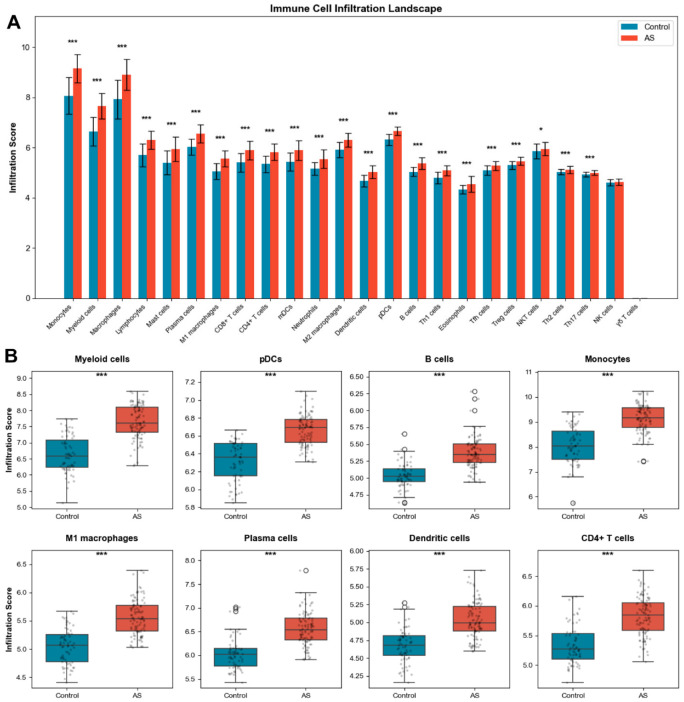
Comparison of immune cell infiltration between AS and control samples in the ComBat-corrected training cohort. Immune cell infiltration scores were estimated using single-sample gene set enrichment analysis (ssGSEA). (**A**) Comparison of infiltration scores for 24 immune cell types between the two groups. Data are presented as mean ± standard deviation (SD). (**B**) Boxplots showing the infiltration scores of eight representative immune cell types with significant differences between the AS and control groups, including myeloid cells, plasmacytoid dendritic cells (pDCs), B cells, monocytes, M1 macrophages, plasma cells, dendritic cells, and CD4^+^ T cells. Boxes represent the interquartile range (IQR), the center line indicates the median, whiskers represent 1.5 × IQR, and each dot represents an individual sample. * *p* < 0.05, *** *p* < 0.001.

The overall immune infiltration patterns were further summarized in the bar plot, which displayed the mean infiltration scores of all 24 immune cell types (Figure 10A). Differential analysis demonstrated that 22 of the 24 immune cell types showed significantly increased infiltration in the AS group after Benjamini–Hochberg false discovery rate (FDR) correction (FDR < 0.05). For clarity, boxplots of eight representative immune cell types showing significant differences between the two groups are presented (Figure 10B). Among all differentially infiltrated immune cell types, monocytes (log_2_FC = 1.084), myeloid cells (log_2_FC = 1.015), and macrophages (log_2_FC = 0.985) exhibited the largest increases in infiltration levels. In contrast, NK cells showed no statistically significant difference between the AS and Control groups. Detailed statistical results, including the mean infiltration scores, standard deviations, log_2_FC values, and FDR-adjusted *p* values for all 24 immune cell types, are provided in Supplementary Table S7. The top 10 differentially infiltrated immune cells with the largest effect sizes are summarized in Table 4.

**Figure 11:**
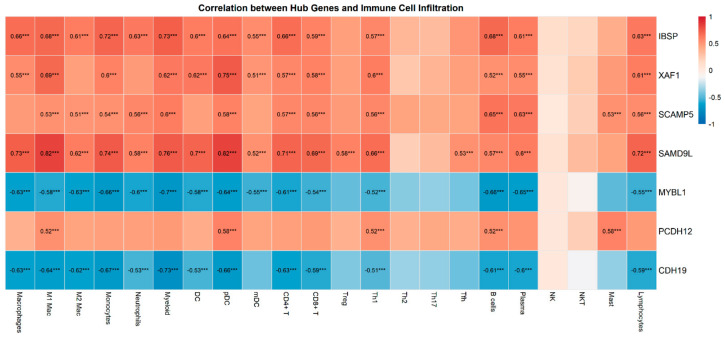
**Correlation between hub genes and immune cell infiltration.** Spearman correlation analysis was performed to evaluate the associations between the expression levels of the seven hub genes and the infiltration scores of 24 immune cell types estimated by single-sample gene set enrichment analysis (ssGSEA). The color scale represents the Spearman correlation coefficient (ρ), with red indicating positive correlations and blue indicating negative correlations. Correlation coefficients are displayed in the heatmap. Only correlations with |ρ| ≥ 0.5 are shown. *** indicates *p* < 0.001.

The relationships between the seven hub genes and immune cell infiltration were subsequently evaluated using Spearman correlation analysis (Figure 11). SAMD9L exhibited the strongest positive correlations with M1 macrophages (ρ = 0.823) and plasmacytoid dendritic cells (pDCs; ρ = 0.821). IBSP was strongly positively correlated with myeloid cells (ρ = 0.735), monocytes (ρ = 0.716), and macrophages (ρ = 0.664). Conversely, MYBL1 and CDH19 showed significant negative correlations with multiple immune cell populations, whereas PCDH12 displayed moderate positive correlations with selected immune cell subsets. The major correlations are summarized in Table 5, and the complete correlation matrix is provided in Supplementary Table S8. These findings indicate that the identified hub genes are closely associated with immune cell infiltration and the inflammatory immune microenvironment in atherosclerosis.

## 4. Discussion

AS is a chronic inflammatory disease characterized by complex interactions between lipid metabolism, immune responses, and vascular remodeling. In this study, we integrated two independent gene expression datasets and performed batch effect correction using the ComBat algorithm to improve data comparability. Differential expression analysis identified 1349 DEGs between atherosclerosis and control samples. To explore the biological relevance of these genes, functional enrichment analysis was performed, revealing that immune- and inflammation-related pathways were significantly enriched. Subsequently, a combination of LASSO regression and Random Forest analysis was applied to identify robust diagnostic biomarkers, resulting in the selection of seven hub genes, including IBSP, XAF1, SCAMP5, SAMD9L, MYBL1, PCDH12, and CDH19.

A multivariable logistic regression model based on these seven genes demonstrated excellent diagnostic performance in the training cohort, with strong discrimination ability and stable cross-validation results. Furthermore, three independent external validation cohorts confirmed the robustness and generalizability of the model. Finally, immune infiltration analysis revealed significant alterations in the immune microenvironment of atherosclerosis, and correlation analysis suggested close associations between hub genes and immune cell infiltration. Overall, these findings provide a multi-level understanding of the molecular characteristics of atherosclerosis and highlight potential diagnostic biomarkers and immune-related mechanisms involved in disease progression.

Functional enrichment analysis demonstrated that the identified DEGs were predominantly involved in immune- and inflammation-related biological processes, highlighting the central role of immune dysregulation in AS. GO analysis showed significant enrichment in leukocyte activation, leukocyte migration, T-cell activation, and leukocyte-mediated immunity, suggesting that abnormal immune cell recruitment and activation are major pathological characteristics of atherosclerotic lesions. These findings are consistent with previous studies demonstrating that persistent leukocyte infiltration and chronic vascular inflammation contribute to endothelial dysfunction, foam cell formation, and plaque progression [31].

KEGG pathway analysis further supported this conclusion. Several significantly enriched pathways, including the chemokine signaling pathway, phagosome, cell adhesion molecules (CAMs), lysosome, and osteoclast differentiation, have been implicated in the development of atherosclerosis. Chemokine signaling regulates the recruitment and migration of circulating monocytes and lymphocytes into the arterial wall, thereby initiating and sustaining vascular inflammation [31]. Cell adhesion molecules facilitate leukocyte adhesion to activated endothelial cells, representing a critical early event during plaque formation [32,33]. Meanwhile, phagosome- and lysosome-related pathways are closely associated with macrophage phagocytosis, lipid metabolism, and foam cell formation [34,35]. In addition, the osteoclast differentiation pathway reflects inflammatory activation of myeloid-derived cells and has been associated with vascular calcification in atherosclerosis [36].

Collectively, these enrichment results indicate that inflammatory activation, immune cell recruitment, and innate immune responses represent fundamental biological processes underlying AS, providing biological support for the subsequent identification of immune-related diagnostic biomarkers.

The present study identified seven hub genes, namely IBSP, XAF1, SCAMP5, SAMD9L, MYBL1, PCDH12, and CDH19, through the combined application of LASSO regression and Random Forest analysis. Compared with using a single machine learning algorithm, this integrated strategy reduced feature redundancy and improved the robustness of biomarker selection, ultimately yielding a compact diagnostic signature with excellent predictive performance in both the training cohort and three independent validation cohorts.

Among these hub genes, IBSP demonstrated particularly strong diagnostic performance and exhibited close associations with immune cell infiltration, especially monocytes, macrophages, and myeloid cells. Although IBSP has traditionally been recognized as a bone matrix protein involved in osteogenesis and mineralization, accumulating evidence indicates that it also participates in vascular calcification, extracellular matrix remodeling, and inflammatory responses [37,38,39]. Vascular calcification is an important pathological characteristic of advanced atherosclerotic lesions and is closely associated with plaque instability and adverse cardiovascular events [40]. Therefore, the significant upregulation of IBSP observed in the present study suggests that it may contribute not only to vascular calcification but also to inflammation-mediated plaque progression.

Another notable finding was the significant upregulation of SAMD9L and XAF1, both of which are involved in interferon-mediated immune responses. SAMD9L has been reported to regulate innate immunity and inflammatory signaling, whereas XAF1 functions as a pro-apoptotic factor induced by interferons and has been implicated in immune activation under inflammatory conditions [41,42]. Previous studies have further demonstrated that XAF1 promotes macrophage apoptosis and necrotic core formation during atherosclerosis, suggesting a potential role in plaque progression [43]. The strong positive correlations between SAMD9L and M1 macrophages as well as plasmacytoid dendritic cells observed in the present study further support its potential role in shaping the inflammatory immune microenvironment during AS progression.

In contrast, MYBL1 and CDH19 were significantly downregulated in AS and exhibited predominantly negative correlations with immune cell infiltration. MYBL1 is a transcription factor associated with cell proliferation and differentiation, whereas CDH19 belongs to the cadherin family and contributes to cell–cell adhesion [44,45]. Their decreased expression may reflect disruption of normal vascular homeostasis during chronic inflammation. In addition, SCAMP5 and PCDH12 may participate in vesicle trafficking and endothelial cell function, respectively, although their precise roles in atherosclerosis remain incompletely understood [46,47]. Notably, these relatively understudied genes consistently demonstrated favorable diagnostic performance across multiple independent cohorts and showed close associations with immune cell infiltration, suggesting that they may represent novel molecular candidates involved in AS pathogenesis. Despite the limited evidence regarding the involvement of these genes in atherosclerosis, their reproducible diagnostic performance across three independent validation cohorts and their close associations with immune cell infiltration support their potential as promising biomarker candidates warranting further mechanistic investigation. These findings suggest that the seven hub genes represent multiple biological processes involved in AS rather than a single inflammatory pathway, highlighting the complex molecular mechanisms underlying disease progression.

Immune cell infiltration is increasingly recognized as a hallmark of atherosclerosis and plays a critical role throughout plaque initiation, progression, and destabilization [48]. Consistent with this concept, ssGSEA analysis demonstrated extensive remodeling of the immune microenvironment in AS, characterized by increased infiltration of multiple immune cell populations. In particular, monocytes, macrophages, and myeloid cells exhibited the most pronounced increases, highlighting the importance of innate immune activation during disease progression [49].

Monocytes are recruited from the circulation into the arterial intima, where they differentiate into macrophages and subsequently form lipid-laden foam cells through excessive uptake of modified lipoproteins [50,51]. Activated macrophages further amplify local inflammation by secreting pro-inflammatory cytokines, chemokines, and matrix-degrading enzymes, thereby accelerating plaque growth and promoting plaque instability [52]. The prominent enrichment of monocyte–macrophage-associated immune cells observed in the present study is therefore consistent with the well-established inflammatory characteristics of atherosclerosis.

Correlation analysis further strengthened the biological relevance of the identified hub genes. Among them, SAMD9L exhibited the strongest positive correlations with M1 macrophages and plasmacytoid dendritic cells, suggesting its potential involvement in pro-inflammatory immune activation. Similarly, IBSP showed strong positive correlations with monocytes, macrophages, and myeloid cells, indicating that its increased expression may be closely linked to inflammatory cell recruitment and vascular remodeling. In contrast, MYBL1 and CDH19 demonstrated predominantly negative correlations with immune cell infiltration, implying that their reduced expression may accompany progressive disruption of vascular homeostasis during chronic inflammation.

Taken together, these findings suggest that the seven hub genes are not only diagnostic biomarkers but are also closely associated with immune microenvironment remodeling in AS. The coordinated interactions between inflammatory gene expression and immune cell infiltration may contribute to plaque progression and provide potential therapeutic targets for immunomodulatory intervention.

The present findings may also have important clinical implications. The seven-gene diagnostic signature demonstrated excellent performance in both the training cohort and three independent validation cohorts, suggesting its potential utility for the early identification of individuals with atherosclerosis. Compared with single-gene biomarkers, the multigene model integrates complementary biological information and therefore provides greater diagnostic robustness. Moreover, the close associations between the identified hub genes and immune cell infiltration indicate that these biomarkers may also reflect inflammatory activity within atherosclerotic lesions, providing a potential molecular basis for individualized risk assessment and future immunomodulatory therapeutic strategies. Although the present signature was developed from plaque tissue transcriptomic data, future studies are warranted to evaluate its applicability in minimally invasive clinical specimens, such as peripheral blood, circulating leukocytes, plasma-derived exosomes, or cell-free RNA, before clinical translation can be considered.

It should be noted that disease diagnosis (AS vs. Control) and plaque risk stratification represent related but distinct clinical tasks. Although the external validation cohorts involved different plaque phenotypes, including early versus advanced lesions and stable versus unstable plaques, these conditions share common pathological mechanisms, such as chronic inflammation, immune dysregulation, extracellular matrix remodeling, and macrophage activation. Therefore, the consistent diagnostic performance observed across these independent cohorts suggests that the seven-gene signature captures core molecular alterations associated with atherosclerotic plaque biology rather than dataset-specific transcriptional characteristics. Nevertheless, prospective validation in larger AS versus Control cohorts is still required before clinical application.

Several strengths of the present study should be highlighted. First, unlike many previous transcriptomic studies that directly merged public datasets, batch effects between the two training datasets were removed using the ComBat algorithm prior to downstream analyses. Principal component analysis confirmed effective correction of batch effects, thereby improving the reliability and comparability of the integrated transcriptomic data. Second, hub genes were identified using a combination of LASSO regression and Random Forest analysis, which reduced feature redundancy and improved the robustness of biomarker selection. Third, the diagnostic model demonstrated stable performance through 5-fold cross-validation and was further validated in three independent external cohorts, supporting its robustness and generalizability. Finally, the integration of immune infiltration analysis with hub gene correlation analysis provided additional biological evidence linking the identified biomarkers to immune microenvironment remodeling in AS, thereby improving the biological interpretability of the diagnostic model.

Several limitations should also be acknowledged. First, all analyses were based on publicly available transcriptomic datasets, and prospective validation in large clinical cohorts is still required. Second, although three independent external validation cohorts were included, the overall sample size remained relatively limited. Third, the present study was primarily based on bioinformatics analyses, and the biological functions and molecular mechanisms of the identified hub genes require further experimental validation using in vitro and in vivo models. Finally, although ComBat batch correction and subsequent PCA/PERMANOVA analyses indicated effective removal of batch effects, residual heterogeneity related to differences in patient characteristics, tissue processing, and microarray platforms cannot be completely excluded. Future studies integrating multicenter clinical cohorts and functional experiments will help clarify the translational potential of these biomarkers.

In summary, the present study established a robust inflammation-related diagnostic signature for atherosclerosis by integrating transcriptomic analysis, machine learning, external validation, and immune infiltration analysis. The seven identified hub genes exhibited excellent diagnostic performance and were closely associated with immune microenvironment remodeling. These findings provide new insights into the molecular mechanisms underlying atherosclerosis and identify potential biomarkers that may facilitate early diagnosis and future immunomodulatory therapeutic strategies.

## 5. Conclusions

In conclusion, this study integrated multiple GEO transcriptomic datasets to systematically investigate the molecular characteristics of AS. Functional enrichment analysis demonstrated that the identified differentially expressed genes were predominantly involved in immune- and inflammation-related biological processes and pathways, highlighting the central role of immune dysregulation in AS. Using a combined machine learning strategy based on LASSO regression and Random Forest analysis, seven hub genes (IBSP, XAF1, SCAMP5, SAMD9L, MYBL1, PCDH12, and CDH19) were identified, and a seven-gene logistic regression model demonstrated excellent diagnostic performance in both the training cohort and three independent external validation cohorts.

Furthermore, immune infiltration analysis revealed extensive remodeling of the immune microenvironment in AS, particularly increased infiltration of monocytes, macrophages, and myeloid cells. Correlation analysis further demonstrated close associations between the identified hub genes and immune cell infiltration, suggesting that these biomarkers are closely linked to inflammatory immune remodeling during AS progression. Collectively, these findings provide new insights into the molecular mechanisms underlying AS and identify potential diagnostic biomarkers that may facilitate early diagnosis, risk assessment, and future investigations of immune-related therapeutic strategies.

## Figures and Tables

**Table 1:** **Top 15 differentially expressed genes between AS and Control groups in the training cohort**.

Rank	Gene	log_2_FC	Adj.*p*	Regulation
1	*MMP9*	3.056	5.99 × 10^−23^	Up
2	*IBSP*	2.311	1.08 × 10^−25^	Up
3	*MMP7*	2.234	2.68 × 10^−14^	Up
4	*ACP5*	2.217	3.45 × 10^−19^	Up
5	*MMP12*	2.034	4.88 × 10^−12^	Up
6	*CCL18*	1.933	2.52 × 10^−11^	Up
7	*CHI3L1*	1.926	7.03 × 10^−15^	Up
8	*SPP1*	1.786	7.95 × 10^−13^	Up
9	*CCL3*	1.773	1.92 × 10^−18^	Up
10	*CD36*	1.73	3.46 × 10^−19^	Up
11	*MYOC*	−1.726	3.69 × 10^−16^	Down
12	*HMOX1*	1.69	1.73 × 10^−19^	Up
13	*IGJ*	1.685	3.39 × 10^−14^	Up
14	*CD52*	1.661	4.29 × 10^−20^	Up
15	*PLA2G7*	1.659	9.64 × 10^−16^	Up

Note: Genes were identified using limma with empirical Bayes moderation. DEGs were defined as genes with |log_2_FC| > 0.5 and adjusted *p* < 0.05 (Benjamini–Hochberg FDR correction). Regulation indicates whether the gene was up-regulated or down-regulated in AS samples compared to Control samples.

**Table 2:** **Seven hub genes selected by the intersection of LASSO and Random Forest**.

Rank	Gene	LASSO_Coefficient	RF_Importance	Regulation
1	*IBSP*	0.38	4	Up
2	*XAF1*	0.763	1	Up
3	*SCAMP5*	0.464	3	Up
4	*SAMD9L*	6.50 × 10^−5^	8	Up
5	*MYBL1*	−0.57	2	Down
6	*PCDH12*	0.115	7	Up
7	*CDH19*	−0.295	6	Down

Note: LASSO regression (lambda.1se) and Random Forest feature importance ranking were applied to the DEGs in the training cohort. The final hub genes were defined as the overlapping genes between LASSO-selected candidates and the top-ranking features from Random Forest. LASSO_Coefficient represents the regression coefficient from the LASSO model. RF_Importance indicates the rank of each gene in Random Forest feature importance.

**Table 3:** **Diagnostic performance of the 7-gene model in the training cohort and three independent validation cohorts**.

Dataset	AUC	95% CI	Sensitivity	Specificity	N
Training	0.992	0.981–1.000	0.950	0.970	168
GSE41571	1.000	1.000–1.000	1.000	1.000	11
GSE28829	0.952	0.886–1.000	0.875	0.923	29
GSE120521	1.000	1.000–1.000	1.000	1.000	14

Note: The model was constructed using logistic regression based on the seven hub genes. Sensitivity and specificity were calculated at the optimal cutoff determined by the Youden index. AUC: area under the ROC curve; N: number of samples in each cohort.

**Table 4:** **Top 10 differentially infiltrated immune cells between Control and AS groups in the training cohort**.

Cell_Type	Control_Mean_SD	AS_Mean_SD	log_2_FC	FDR
Monocytes	8.07 ± 0.74	9.15 ± 0.57	1.084	6.47 × 10^−16^
Myeloid cells	6.64 ± 0.57	7.65 ± 0.51	1.015	4.73 × 10^−17^
Macrophages	7.93 ± 0.77	8.91 ± 0.62	0.985	9.57 × 10^−13^
Lymphocytes	5.70 ± 0.45	6.29 ± 0.36	0.592	5.55 × 10^−13^
Mast cells	5.40 ± 0.47	5.94 ± 0.48	0.54	1.42 × 10^−10^
Plasma cells	6.03 ± 0.33	6.56 ± 0.35	0.529	2.17 × 10^−15^
M1 macrophages	5.05 ± 0.31	5.56 ± 0.31	0.517	1.11 × 10^−15^
CD8+ T cells	5.40 ± 0.37	5.89 ± 0.36	0.488	3.03× 10^−12^
CD4+ T cells	5.34 ± 0.33	5.83 ± 0.33	0.485	7.31× 10^−14^
mDCs	5.43 ± 0.36	5.89 ± 0.39	0.462	2.01× 10^−11^

Note: Immune cell infiltration scores were quantified using ssGSEA based on the ComBat corrected expression matrix. Data are presented as mean ± SD. Differences between groups were assessed using the Mann–Whitney U test with Benjamini–Hochberg FDR correction. FDR < 0.05 was considered statistically significant.

**Table 5:** **Spearman correlations between the 7 hub genes and key immune cell types in the training cohort**.

Gene	Macrophages	Monocytes	Myeloid	M1_Mac	pDC	Lymphocytes
*IBSP*	0.664 ***	0.716 ***	0.735 ***	0.677 ***	0.640 ***	0.631 ***
*XAF1*	0.554 ***	0.595 ***	0.616 ***	0.689 ***	0.749 ***	0.605 ***
*SCAMP5*	—	0.535 ***	0.602 ***	0.534 ***	0.584 ***	0.555 ***
*SAMD9L*	0.730 ***	0.744 ***	0.755 ***	0.823 ***	0.821 ***	0.716 ***
*MYBL1*	−0.626 ***	−0.659 ***	−0.697 ***	−0.585 ***	−0.644 ***	−0.553 ***
*PCDH12*	—	—	—	0.521 ***	0.581 ***	—
*CDH19*	−0.633 ***	−0.667 ***	−0.726 ***	−0.644 ***	−0.659 ***	−0.594 ***

Note: The Spearman correlation coefficients (ρ) were calculated between the expression levels of each hub gene and the infiltration scores of six key immune cell types. Only |ρ| ≥ 0.5 and FDR < 0.05 are displayed. *** FDR < 0.001—indicates |ρ| < 0.5.

## Data Availability

The datasets analyzed in this study are available from the Gene Expression Omnibus (GEO) database. The training datasets are GSE100927 and GSE43292, and the external validation datasets are GSE41571, GSE120521, and GSE28829. All datasets are publicly accessible at https://www.ncbi.nlm.nih.gov/geo/ (accessed on 10 May 2026).

## Supplementary Materials

The following supporting information can be downloaded at: https://www.mdpi.com/article/10.3390/genes17070830/s1. Figure S1. Principal component analysis (PCA) of the ComBat-corrected training cohort with samples colored according to biological group (Control vs. AS). (A) Two-dimensional PCA plot. (B) Two-dimensional PCA plot with 95% confidence ellipses. (C) Three-dimensional PCA plot. PC1 and PC2 explained 19.1% and 7.8% of the total variance, respectively. PERMANOVA analysis confirmed significant separation between the Control and AS groups (F = 34.5244, *p* = 0.001, 999 permutations), indicating that biological differences were preserved after batch correction. Figure S2. Venn diagram of differentially expressed genes (DEGs) shared between GSE43292 and GSE100927. DEGs were identified using the criteria of |log_2_FC| > 0.5 and adjusted *p* < 0.05 (Benjamini–Hochberg false discovery rate correction). A total of 1098 DEGs were identified in GSE43292 and 1868 DEGs were identified in GSE100927. Among them, 719 DEGs were shared between the two datasets, representing 65.5% of the DEGs identified in GSE43292. Figure S3. Calibration plot of the 7 gene logistic regression model in the training cohort. The x axis represents the predicted probability of AS, and the y axis represents the observed proportion of AS. The diagonal dashed line indicates perfect calibration. Points represent the mean predicted probability versus the observed proportion within each decile group, and error bars indicate 95% confidence intervals. The calibration curve demonstrates good agreement between predicted and observed probabilities, with points lying close to the diagonal line, indicating that the model is well calibrated. Figure S4. Variable importance ranking of the 16 candidate genes identified by the random forest model. Feature importance was evaluated using the Mean Decrease Gini metric. Genes with higher Mean Decrease Gini values contributed more to the classification performance of the random forest model. Table S1. Summary of the training datasets (GSE43292 and GSE100927) used in this study. The table summarizes the corresponding microarray platforms and the numbers of control and ankylosing spondylitis (AS) samples included in each dataset. In total, the merged training set comprised 168 samples, including 67 control samples and 101 AS samples. Table S2. Gene signatures used for ssGSEA-based immune cell infiltration analysis. The table lists the gene signatures used to define 24 immune cell types in the ssGSEA analysis. Gene signatures were obtained from Charoentong et al. [30]. Gene Count indicates the number of genes included in each signature. Table S3. Summary of statistical methods used in this study. This table summarizes the statistical methods, multiple-testing correction procedures, and software packages applied for each analysis in the study. Abbreviations: CV, cross-validation; FDR, false discovery rate; GO, Gene Ontology; KEGG, Kyoto Encyclopedia of Genes and Genomes; LASSO, least absolute shrinkage and selection operator; ROC, receiver operating characteristic; ssGSEA, single-sample gene set enrichment analysis. Table S4. Summary of DEG overlap analysis between GSE43292 and GSE100927. Differentially expressed genes (DEGs) were identified independently in GSE43292 and GSE100927 using the ComBat-corrected expression data. DEGs were defined as genes with |log_2_FC| > 0.5 and adjusted *p* < 0.05 (Benjamini–Hochberg FDR correction). Among the 17,573 shared genes analyzed, 1098 DEGs were identified in GSE43292 and 1868 DEGs in GSE100927. A total of 719 DEGs overlapped between the two datasets, representing 65.5% of the DEGs identified in GSE43292. Table S5. LASSO-selected 30 candidate genes with non-zero coefficients. The LASSO regression model with five-fold cross-validation identified 30 genes with non-zero coefficients at the optimal λ value. Positive coefficients indicate upregulated genes (higher expression in AS), whereas negative coefficients indicate downregulated genes (lower expression in AS). These 30 genes were used for subsequent Random Forest analysis. Table S6. Random Forest feature importance of the 16 LASSO-selected candidate genes. MeanDecreaseAccuracy and MeanDecreaseGini represent the contribution of each gene to the Random Forest model. Higher values indicate greater importance in distinguishing AS from Control samples. Table S7. Complete immune cell infiltration analysis of 24 cell types between Control and AS groups. Immune cell infiltration scores were quantified using ssGSEA based on the ComBat-corrected expression matrix. Data are presented as mean ± SD. Differences between Control and AS groups were assessed using the Mann–Whitney U test, with *p* values adjusted using the Benjamini–Hochberg false discovery rate (FDR) correction. TRUE indicates FDR < 0.05 (statistically significant). γδ T cells showed no detectable enrichment in the training cohort. Table S8. Spearman correlations between 7 hub genes and 24 immune cell types. Spearman correlation coefficients (ρ) between the expression levels of the seven hub genes and the infiltration scores of 24 immune cell types are shown. Only correlations with |ρ| ≥ 0.5 and FDR < 0.05 are displayed. *** indicates FDR < 0.001. Empty cells indicate correlations with |ρ| < 0.5 or FDR ≥ 0.05. γδ T cells showed no detectable infiltration signal in the training cohort.

## Abbreviations

AS, Atherosclerosis; DEGs, Differentially expressed genes; GO, Gene Ontology; KEGG, Kyoto Encyclopedia of Genes and Genomes; LASSO, Least absolute shrinkage and selection operator; ROC, Receiver operating characteristic; AUC, Area under the curve; CI, Confidence interval; GEO, Gene Expression Omnibus; PCA, Principal component analysis; PERMANOVA, Permutational multivariate analysis of variance; MA, M–A plot; BP, Biological process; CC, Cellular component; MF, Molecular function; FDR, False discovery rate; NK, Natural killer cells; IBSP, Integrin-binding sialoprotein; XAF1, XIAP-associated factor 1; SCAMP5, Secretory carrier membrane protein 5; SAMD9L, Sterile alpha motif domain containing 9-like; MYBL1, MYB proto-oncogene like 1; PCDH12, Protocadherin 12; CDH19, Cadherin 19; SNORD116-4, Small nucleolar RNA, C/D box 116-4; APOC1, Apolipoprotein C-I; MOB3A, MOB kinase activator 3A; CLTC-IT1, Clathrin heavy chain intronic transcript 1; HLA-DOB, Major histocompatibility complex, class II, DO beta; SERPINA3, Serpin family A member 3; CX3CR1, C-X3-C motif chemokine receptor 1; C4B, Complement C4B; GPC3, Glypican 3; NR1D1, Nuclear receptor subfamily 1 group D member 1.
