# Supplementary material for: Integrated Bioinformatics and Machine Learning Analysis Identifies Inflammation-Related Biomarkers and Immune Infiltration Patterns in Atherosclerosis
Source: Genes (Basel). 2026 Jul 21;17(7):830. doi: 10.3390/genes17070830 (PMC13409607; doi:10.3390/genes17070830)
Supplement: Supplementary file 1 [file genes-17-00830-s001.zip › genes-4375960-supplementary.pdf]

Figure S1. Principal component analysis (PCA) of the merged training datasets after ComBat batch effect correction.

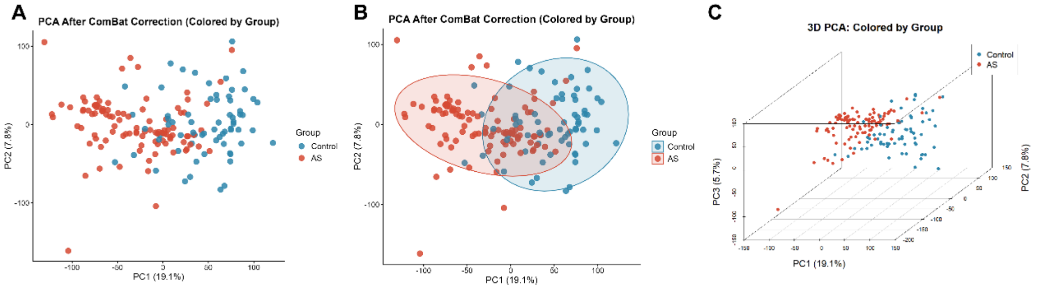

Figure S1. Principal component analysis (PCA) of the ComBat-corrected training cohort with samples colored according to biological group (Control vs. AS). (A) Two-dimensional PCA plot. (B) Two-dimensional PCA plot with 95% confidence ellipses. (C) Three-dimensional PCA plot. PC1 and PC2 explained 19.1% and 7.8% of the total variance, respectively. PERMANOVA analysis confirmed significant separation between the Control and AS groups (F = 34.5244, P = 0.001, 999 permutations), indicating that biological differences were preserved after batch correction.

Figure S2. Venn diagram of differentially expressed genes (DEGs) shared between GSE43292 and GSE100927.

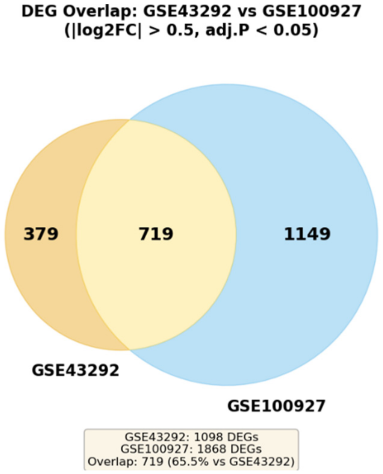

Figure S2. Venn diagram of differentially expressed genes (DEGs) shared between GSE43292 and GSE100927. DEGs were identified using the criteria of $|\log_2 FC| > 0.5$ and adjusted $P < 0.05$ (Benjamini–Hochberg false discovery rate correction). A total of 1,098 DEGs were identified in GSE43292 and 1,868 DEGs were identified in GSE100927. Among them, 719 DEGs were shared between the two datasets, representing 65.5% of the DEGs identified in GSE43292.

Figure S3. Calibration plot of the 7-gene logistic regression model in the training cohort.

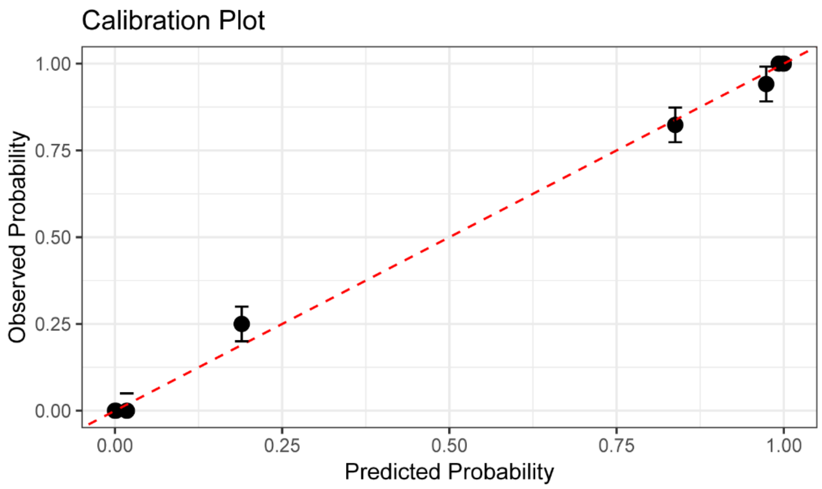

Figure S3. The x-axis represents the predicted probability of AS, and the y-axis represents the observed proportion of AS. The diagonal dashed line indicates perfect calibration. Points represent the mean predicted probability versus the observed proportion within each decile group, and error bars indicate 95% confidence intervals. The calibration curve demonstrates good agreement between predicted and observed probabilities, with points lying close to the diagonal line, indicating that the model is well-calibrated.

Figure S4. Variable importance ranking of the 16 candidate genes identified by the random forest model.

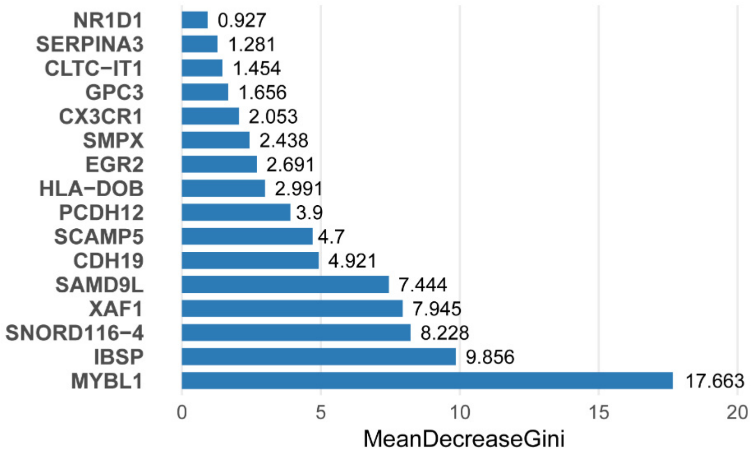

Figure S4. Feature importance was evaluated using the Mean Decrease Gini metric. Genes with higher Mean Decrease Gini values contributed more to the classification performance of the random forest model.

Table S1. Summary of the training datasets (GSE43292 and GSE100927) used in this study.

Dataset	Platform	Control	AS	Total
GSE43292	GPL6244	32	32	64
GSE100927	GPL17077	35	69	104
Total		67	101	168

Table S1. The table summarizes the corresponding microarray platforms and the numbers of control and ankylosing spondylitis (AS) samples included in each dataset. In total, the merged training set comprised 168 samples, including 67 control samples and 101 AS samples.

Table S2. Gene signatures used for ssGSEA based immune cell infiltration analysis.

Cell Type	Gene Signatures	Gene Count
B cells	CD19, MS4A1, CD79A, CD79B, CR2, CD22, BLK, CD24, CD37	9
CD4+ T cells	CD4, IL2RA, CTLA4, CD40LG, ICOS, TNFRSF4, TNFRSF18, BATF	8
CD8+ T cells	CD8A, CD8B, GZMA, GZMB, PRF1, EOMES, TBX21, KLRG1	8
Treg cells	FOXP3, IL2RA, CTLA4, IKZF2, TNFRSF18, LEPR, TIGIT	7
NK cells	NCAM1, KIR2DL1, KIR2DL3, KIR3DL1, KLRD1, NCR1, NCR3, FGFBP2	8
NKT cells	CD1D, KLRB1, TRAV10, TRAJ18, CXCR6, ZBTB16	6
Macrophages	CD68, CD163, MSR1, MARCO, TLR2, TLR4, FCGR1A, FCGR2A	8
M1 macrophages	NOS2, IL12A, IL23A, TNF, CXCL10, CXCL11, CCL5	7
M2 macrophages	CD163, CD206, ARG1, IL10, CCL17, CCL22, TGFB1	7
Dendritic cells	FCER1A, CD1A, CD1C, CD1E, CLEC4A, CLEC4C, IDO1	7
pDCs	CLEC4C, IL3RA, IRF7, TCF4, TLR7, TLR9, BATF3, JCHAIN	8
mDCs	CD1A, CD1C, CD1E, CD83, CCL19, CCL21	6

Monocytes	CD14, FCGR3A, CD16, CSF1R, LYZ, S100A8, S100A9, TREM1	8
Neutrophils	FCGR3B, FCGR2A, CD66b, CEACAM8, CXCR2, IL8, ELANE, MPO	8
Eosinophils	IL5RA, CCR3, PRG2, RNASE2, RNASE3, CLC, EPX, SIGLEC8	8
Mast cells	TPSAB1, TPSB2, CPA3, MS4A2, KIT, FCER1A, HDC	7
Th1 cells	IFNG, IL12RB2, TBX21, STAT4, IL18R1, CXCR3	6
Th2 cells	IL4, IL5, IL13, STAT6, GATA3, IL4R, CCR4	7
Th17 cells	IL17A, IL17F, IL22, RORC, STAT3, IL23R, CCR6	7
Tfh cells	BCL6, IL21, CXCR5, PDCD1, ICOS, SAP, SH2D1A	7
γδ T cells	TRGC1, TRGC2, TRDV1, TRDV2, CLEC2F, VSTM1, BTN3A1	7
Plasma cells	SDC1, XBP1, IRF4, PRDM1, CD38, BLIMP1, POU2AF1	7
Lymphocytes	PTPRC, CD2, CD3D, CD3E, CD3G, CD247, LCK, ZAP70	8
Myeloid cells	CD33, ITGAM, ITGAX, CSF1R, MPO, LYN, SYK	7

Table S2. Gene signatures used for ssGSEA-based immune cell infiltration analysis. The table lists the gene signatures used to define 24 immune cell types in the ssGSEA analysis. Gene signatures were obtained from Charoentong et al. {Charoentong, 2017 #108}. Gene Count indicates the number of genes included in each signature.

Table S3. Summary of statistical methods used in this study.

Analysis	Statistical Method	Correction	Software
Differential expression analysis	limma (moderated t-test)	Benjamini-Hochberg FDR	limma (R)
GO/KEGG enrichment analysis	Fisher's exact test	Benjamini-Hochberg FDR	clusterProfiler (R)
LASSO feature selection	L1-regularized regression with 5-fold CV	—	glmnet (R)
Random Forest feature selection	Random Forest	—	randomForest (R)
Logistic regression modeling	Maximum likelihood estimation	—	glm (R)
Model evaluation	ROC curve and AUC analysis	—	pROC (R)
Cross-validation	Stratified 5-fold cross-validation	—	caret (R)
Group comparison (immune cells)	Mann-Whitney U test	Benjamini-Hochberg FDR	stats (R)
Correlation analysis	Spearman rank correlation	Benjamini-Hochberg FDR	stats (R)
Immune infiltration	ssGSEA	—	GSVA (R)

Table S3. Summary of statistical methods used in this study. This table summarizes the statistical methods, multiple-testing correction procedures, and software packages applied for each analysis in the study. Abbreviations: CV, cross-validation; FDR, false discovery rate; GO, Gene Ontology; KEGG, Kyoto Encyclopedia of Genes and Genomes; LASSO, least absolute shrinkage and selection operator; ROC, receiver operating characteristic; ssGSEA, single-sample gene set enrichment analysis.

Table S4. Summary of DEG overlap analysis between GSE43292 and GSE100927.

Item	Value
Shared genes analyzed	17573
DEGs in GSE43292	1098
DEGs in GSE100927	1868
Overlapping DEGs	719
Overlap ratio (vs GSE43292)	65.50%

Table S4. Differentially expressed genes (DEGs) were identified independently in GSE43292 and GSE100927 using the ComBat-corrected expression data. DEGs were defined as genes with $|\log_2 FC| > 0.5$ and adjusted $P < 0.05$ (Benjamini–Hochberg FDR correction). Among the 17,573 shared genes analyzed, 1,098 DEGs were identified in GSE43292 and 1,868 DEGs in GSE100927. A total of 719 DEGs overlapped between the two datasets, representing 65.5% of the DEGs identified in GSE43292.

Table S5. LASSO-selected 30 candidate genes with non-zero coefficients.

Gene	Coefficient	Regulation
APOC1	-0.893	Down
MOB3A	0.833	Up
MYBL1	-0.79	Down
XAF1	0.706	Up
SCAMP5	0.674	Up
SAMD9L	0.576	Up
CDH19	-0.502	Down
CLTC-IT1	0.451	Up
HLA-DOB	0.424	Up
IBSP	0.359	Up
SERPINA3	-0.341	Down
SNORD116-4	-0.315	Down
CX3CR1	0.286	Up
C4B	0.26	Up
GPC3	-0.247	Down
NR1D1	-0.157	Down
DES	0.154	Up
EGR2	0.137	Up
DUSP6	0.131	Up

ACTA1	0.124	Up
HAMP	-0.11	Down
ANPEP	0.061	Up
PCDH12	0.037	Up
TNF	0.024	Up
ICAM3	0.022	Up
C11orf96	0.021	Up
COL4A5	0.02	Up
HBA2	0.015	Up
PCP4	0.007	Up
IFIH1	0.006	Up

Table S5. LASSO-selected 30 candidate genes with non-zero coefficients. The LASSO regression model with five-fold cross-validation identified 30 genes with non-zero coefficients at the optimal λ value. Positive coefficients indicate upregulated genes (higher expression in AS), whereas negative coefficients indicate downregulated genes (lower expression in AS). These 30 genes were used for subsequent Random Forest analysis.

Table S6. Random Forest feature importance of the 16 LASSO-selected candidate genes.

Gene	MeanDecreaseAccuracy	MeanDecreaseGini
MYBL1	21.322	17.663
XAF1	16.645	7.945
IBSP	12.545	9.856
SAMD9L	12.495	7.444
SCAMP5	11.792	4.7
PCDH12	9.916	3.9
CDH19	10.363	4.921
HLA-DOB	10.006	2.991
SMPX	10.156	2.438
CX3CR1	8.31	2.053
SNORD116-4	14.774	8.228
EGR2	10.317	2.691
GPC3	7.303	1.656
CLTC-IT1	7.577	1.454

SERPINA3	7.429	1.281
NR1D1	1.548	0.927

Table S6. Random Forest feature importance of the 16 LASSO-selected candidate genes. MeanDecreaseAccuracy and MeanDecreaseGini represent the contribution of each gene to the Random Forest model. Higher values indicate greater importance in distinguishing AS from Control samples.

Table S7. Complete immune cell infiltration analysis of 24 cell types between Control and AS groups.

Cell Type	Control Mean	Control SD	AS Mean	AS SD	log2FC	FDR	Significant
B cells	5.04	0.184	5.373	0.234	0.333	5.05E-16	TRUE
CD4+ T cells	5.341	0.329	5.826	0.328	0.485	7.00E-14	TRUE
CD8+ T cells	5.404	0.374	5.892	0.363	0.488	2.90E-12	TRUE
Treg cells	5.304	0.157	5.467	0.166	0.163	5.15E-09	TRUE
NK cells	4.607	0.125	4.628	0.129	0.022	2.64E-01	FALSE
NKT cells	5.854	0.293	5.95	0.266	0.096	9.71E-03	TRUE
Macrophages	7.927	0.771	8.913	0.617	0.985	9.17E-13	TRUE
M1 macrophages	5.048	0.313	5.565	0.311	0.517	1.06E-15	TRUE
M2 macrophages	5.912	0.306	6.301	0.279	0.389	1.39E-12	TRUE
Dendritic cells	4.676	0.236	5.032	0.254	0.356	5.62E-14	TRUE
pDCs	6.316	0.219	6.663	0.17	0.347	5.49E-17	TRUE
mDCs	5.433	0.358	5.895	0.386	0.462	1.93E-11	TRUE
Monocytes	8.071	0.735	9.155	0.566	1.084	6.20E-16	TRUE
Neutrophils	5.16	0.261	5.549	0.371	0.39	7.04E-13	TRUE
Eosinophils	4.331	0.164	4.549	0.315	0.218	1.56E-07	TRUE
Mast cells	5.399	0.473	5.939	0.483	0.54	1.36E-10	TRUE
Th1 cells	4.804	0.236	5.09	0.206	0.286	1.87E-12	TRUE
Th2 cells	5.03	0.1	5.119	0.141	0.089	7.84E-06	TRUE
Th17 cells	4.929	0.095	5	0.104	0.071	1.45E-05	TRUE
Tfh cells	5.094	0.187	5.278	0.189	0.184	8.90E-09	TRUE
γδ T cells	0	0	0	0	0	NaN	NA
Plasma cells	6.028	0.328	6.557	0.352	0.529	2.08E-15	TRUE
Lymphocytes	5.703	0.454	6.295	0.361	0.592	5.32E-13	TRUE

Myeloid cells	6.639	0.568	7.654	0.513	1.015	4.53E-17	TRUE

Table S7. Complete immune cell infiltration analysis of 24 cell types between Control and AS groups. Immune cell infiltration scores were quantified using ssGSEA based on the ComBat-corrected ex-pression matrix. Data are presented as mean ± SD. Differences between Control and AS groups were assessed using the Mann–Whitney U test, with P values adjusted using the Benjamini–Hochberg false discovery rate (FDR) correction. TRUE indicates FDR < 0.05 (statistically significant). γδ T cells showed no detectable enrichment in the training cohort.

Table S8. Spearman correlations between 7 hub genes and 24 immune cell types.

Gene	IBSP	XAF1	SCAMP5	SAMD9L	MYBL1	PCDH12	CDH19
B cells	0.682 ***	0.519 ***	0.655 ***	0.573 ***	-0.659 ***	0.524 ***	-0.611 ***
CD4+ T cells	0.658 ***	0.566 ***	0.574 ***	0.712 ***	-0.608 ***	—	-0.634 ***
CD8+ T cells	0.593 ***	0.583 ***	0.563 ***	0.693 ***	-0.536 ***	—	-0.589 ***
Treg cells	—	—	—	0.578 ***	—	—	—
NK cells	—	—	—	—	—	—	—
NKT cells	—	—	—	—	—	—	—
Macrophages	0.664 ***	0.554 ***	—	0.73 ***	-0.626 ***	—	-0.633 ***
M1 macrophages	0.677 ***	0.689 ***	0.534 ***	0.823 ***	-0.585 ***	0.521 ***	-0.644 ***
M2 macrophages	0.612 ***	—	0.509 ***	0.615 ***	-0.634 ***	—	-0.624 ***
Dendritic cells	0.597 ***	0.621 ***	—	0.7***	-0.58 ***	—	-0.534 ***
pDCs	0.64 ***	0.749 ***	0.584 ***	0.821 ***	-0.644 ***	0.581 ***	-0.659 ***
mDCs	0.548 ***	0.506 ***	—	0.519 ***	-0.548 ***	—	—
Monocytes	0.716 ***	0.595 ***	0.535 ***	0.744 ***	-0.659 ***	—	-0.667 ***
Neutrophils	0.631 ***	—	0.559 ***	0.583 ***	-0.598 ***	—	-0.534 ***
Eosinophils	—	—	—	—	—	—	—
Mast cells	—	—	0.526 ***	—	—	0.577 ***	—

Th1 cells	0.568 ***	0.604 ***	0.561 ***	0.658 ***	-0.518 ***	0.518 ***	-0.509 ***
Th2 cells	—	—	—	—	—	—	—
Th17 cells	—	—	—	—	—	—	—
Tfh cells	—	—	—	0.53 ***	—	—	—
γδ T cells	—	—	—	—	—	—	—
Plasma cells	0.61 ***	0.547 ***	0.628 ***	0.605 ***	-0.646 ***	0.5 ***	-0.598 ***
Lymphocytes	0.631 ***	0.605 ***	0.555 ***	0.716 ***	-0.553 ***	—	-0.594 ***
Myeloid cells	0.735 ***	0.616 ***	0.602 ***	0.755 ***	-0.697 ***	—	-0.726 ***

Table S8. Spearman correlations between 7 hub genes and 24 immune cell types. Spearman correlation coefficients (ρ) between the expression levels of the seven hub genes and the infiltration scores of 24 immune cell types are shown. Only correlations with $|\rho| \geq 0.5$ and FDR < 0.05 are displayed. *** indicates FDR < 0.001. Empty cells indicate correlations with $|\rho| < 0.5$ or FDR ≥ 0.05. γδ T cells showed no detectable infiltration signal in the training cohort.
